# Current Trends in the Biosensors for Biological Warfare Agents Assay

**DOI:** 10.3390/ma12142303

**Published:** 2019-07-18

**Authors:** Miroslav Pohanka

**Affiliations:** Faculty of Military Health Sciences, University of Defense, Trebesska 1575, CZ-50001 Hradec Kralove, Czech Republic; miroslav.pohanka@gmail.com

**Keywords:** anthrax, *Bacillus anthracis*, bioassay, biological warfare agent, biological weapon, biosensor, colorimetry, electrochemistry, hand held assay, hemorrhagic fever, tularemia

## Abstract

Biosensors are analytical devices combining a physical sensor with a part of biological origin providing sensitivity and selectivity toward analyte. Biological warfare agents are infectious microorganisms or toxins with the capability to harm or kill humans. They can be produced and spread by a military or misused by a terrorist group. For example, *Bacillus anthracis*, *Francisella tularensis*, *Brucella* sp., *Yersinia pestis*, staphylococcal enterotoxin B, botulinum toxin and orthopoxviruses are typical biological warfare agents. Biosensors for biological warfare agents serve as simple but reliable analytical tools for the both field and laboratory assay. There are examples of commercially available biosensors, but research and development of new types continue and their application in praxis can be expected in the future. This review summarizes the facts and role of biosensors in the biological warfare agents’ assay, and shows current commercially available devices and trends in research of the news. Survey of actual literature is provided.

## 1. Introduction

Weapons of mass destruction are devices produced for military or terrorist purposes and their use can cause a large number of victims, or a broad impact on economy and material, or endanger other aspects of human life. The mass destruction weapons are commonly abbreviated as CBRN, deriving from the different types of the main mass destruction weapons: chemical, biological, radiological and nuclear. Compared to other types of mass destruction weapons, biological weapons were not widely used in real, large scale combat such as the other mass destruction weapons. In comparison, chemical weapons were widely applied in e.g., the battlefields of World War I or Iran-Iraq War and the nuclear war, which hastened Japan in the end of World War II to the capitulation. 

Biological weapons are specific types of weapons as most of them are not able to immediately harm and their impact is visible after an incubation period. Countermeasures against the impact of biological weapons on humans is based on contemporary steps, including detection and identification of the used agents by its direct recognition, or by a diagnostic procedure using biological samples from patients, decontamination and providing medical therapy to the infected or poisoned people. This review is focused on biosensors; devices combining physical sensors with a part of biological origin, which are suitable for the detection of biological warfare agents. Biosensors are typically small, portable devices with good applicability in field conditions. This review summarizes expected applicability and parameters of biosensors for the biological warfare agents’ assay. Current literature is surveyed and the expected use of biosensors in context of the standard analytical protocols is provided as well.

## 2. Biological Weapon and Biological Warfare Agents

Although the terms biological weapon and biological warfare agent can appear as synonyms—the contrary is also true. A biological weapon is a mean applicable for military or other warfare purposes, and it contains functional parts necessary for the stabilization and delivery of a harmful organism or its toxin. A biological warfare agent is simply a synonym for the organism or its toxin and infectious cells, viral particles and toxins able to cause infection to humans and livestock, and they cause damage to crops or can poison organisms [1,2,3,4]. Various spray making devices, weak explosives, pressure vessels and etc., can serve as parts for the delivery of biological warfare agents. The weapon can be shaped as a standard projectile or bomb, but standard cases can also be expected in the environment of an asymmetric war or terrorist attacks.

The decision of what is considered a biological warfare agent was made on the international level and was codified as an international convention called: “The Convention on the Prohibition of the Development, Production and Stockpiling of Bacteriological (Biological) and Toxin Weapons and on their Destruction”, also known under the shorter name of the “Biological Weapons Convention”. The convention was signed by most of the countries in the world in 1972—apart from some exceptions—and it came into force in 1975. Biological warfare agents and biological weapons have not been legally produced and stockpiled from the time of the convention entering into force. In a brief summary, the biological warfare agents can be legally produced and manipulated for the purpose of medical or other protective research where therapies, new drugs, decontamination means and etc., are developed. Any offensive research programs are banned by the convention.

It can be expected that every infectious organism can be misused for criminal or warfare activities; on the other hand, some of them are less dangerous and real use is not probable. The reason why some biological agents are a more relevant threat than others is based not only on their virulence or toxicity, but also on the stability of the agent in the environment, how they can penetrate the host organism, etc. The threat from the individual agents was scaled by the Centers for Disease Control and Prevention (CDC; Atlanta, GA, USA) which uses the letters A, B and C for the designation of biological warfare agents in compliance with their level of danger. The lowest threat can be expected from the C category of biological warfare agents, who can endanger a wide population only under some circumstances, and their dissemination is not easy. More serious are biological warfare agents of the B category such as: *Brucella* family (*Brucella melitensis* and *Brucella abortus* causing brucellosis can be exampled), *Clostridium perfringens*, *Salmonella* family, *Schigella* family (agents causing shigellosis), *Escherichia coli* O157:47 (an agent producing shiga toxin and causing foodborne illnesses), *Burkholderia mallei* (a causative agent of glanders) and *Burkholderia psedomallei* (a causative agent of melioidosis), *Chlamydia psittaci* (an agent causing chlamydiosis), *Coxiella burnetti* (a causative agent of Q fever), *Rickettsia prowazekii* (a causative agent of typhus), *Vibrio cholera* (a causative agent of cholera), viruses causing encephalitis, staphylococcal toxins (staphylococcal enterotoxin B for instance) and ricin toxin (a toxin from plant *Ricinus communis*). The biological agents of the B category can be disseminated moderately easy, or they will have a moderate impact on humans. The top dangerous biological warfare agents are given into the A category containing *Bacillus anthracis* (a causative agent of anthrax), *Clostridium botulinum* as well as its botulinum toxin (a group of eight toxins A, B, C, D, E, F, G, H), *Francisella tularensis* (a causative agent of tularemia), *Yersinia pestis* (a causative agent of plaque), and a group of highly virulent viruses (Variola major causing smallpox, viruses of hemorrhagic fevers Ebola, Marburg, Lassa, Machupo). An overview of important biological warfare agents can be found in Table 1. 

## 3. Expected Use of Biosensors during a Biological Threat

There is a lot of highly competitive methods available for the identification of biological warfare agents. The analytical methods for the recognition of biological warfare agent the presence or a diagnosis of diseases caused by them, are necessary for the choice of adequate countermeasures and to start an effective therapy for exposed people [5,6,7]. Mass spectrometry alone or in combination with chromatography are universal tools for the purpose of specific structures identification and are suitable for the determination of viral, bacterial and toxin agents [8,9,10,11,12]. Applicability for the identification of chemical agents by the same equipment is another advantage of mass spectrometry [13,14].

Polymerase chain reaction (PCR) is another method that proved reliability for the purpose of identifying biological warfare agents. It is not suitable for proving that a toxic product comes from biological entities, but it typically has typically excellent sensitivity to viruses and bacteria. Theoretically, only one cell or virion particle can be identified by PCR. Real performances of PCR provide limits of detection in the level of dozens microorganisms such as exerted in the work by Saikaly and coworkers [15] for the assay of *Serratia marcescens* and *Bacillus atrophaeus* representing surrogates for *Bacillus anthracis* and *Yersinia pestis*. The PCR is dependent on used primers, which are responsible for the specificity of the method. Various configurations of the method are available from which real time PCR is likely the most widely used in the current praxis [16,17]. There are also commercially available PCR devices suitable for field assay. 

The mentioned mass spectrometry and PCR are of course not the only methods available for the determination of microorganisms and toxins. A wide number of protocols and methods are available; including cultivation kits, immunochemical assays, laser techniques for biological aerosols, etc. Small hand-held analyzers are very popular devices, and they have place in first response teams. Biosensors and similar bioassays are also very popular in this regard. In exact terminology, a device containing a biorecognition part such as an enzyme or antibody and a sensor—also known as a physical-chemical transducer—can be entitled a biosensor [18,19,20,21,22,23]. Biosensors are not direct competitors to standard and more expensive laboratory methods, but they represent a simple and cheap tool taking place in field analyses, confirming data from the standard laboratory methods or screening samples before selection of the suspicious one. The general role of biosensors in assay of biological warfare agents was also extensively reviewed in the cited papers [24,25,26].

## 4. Optical Biosensors for Biological Warfare Agents Assay

**Figure 1:**
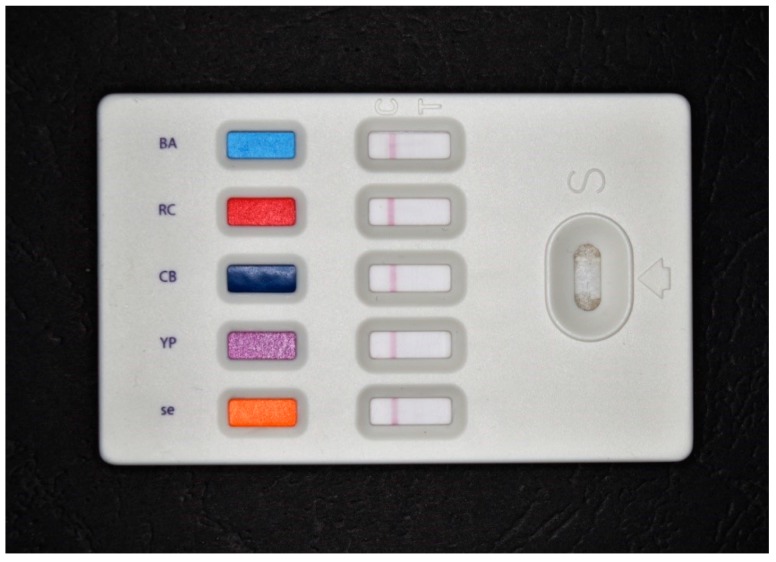
Example of a colorimetric, commercially available, biosensor on the principle of lateral flow assay for the determination of *Bacillus anthracis* (indicated by letters BA on the biosensor), *ricin* (indicated by letters “RC”), *botulinum toxin* (indicated by letters “CB”), *Yersinia pestis* (indicated by letters “YP”)*, staphylococcal enterotoxin B* (indicated by letters “se”).

Optical biosensors and similar bioassays are an extensively researched group of analytical devices, as seen from the recent reviews written in this field [27,28,29,30,31,32,33,34,35,36,37,38]. The optical biosensors also have a long tradition in teams protecting from biological warfare agents because there is a high number of disposable colorimetric tests and portable devices available in the market. The hand-held test strips typically work on the principle of lateral flow (immunochromatography) assay. No instrumentation is necessary for their performance and the strips are suitable for measurement of *Bacillus anthracis*, *Francisella tularensis*, *Brucella* sp., *Yersinia pestis*, staphylococcal enterotoxin B, botulinum toxin and orthopoxviruses. The strips are made for one, five or eight biological warfare agents contemporary measurable in one step. Manufacturers such as Advnt Biotechnologies (Phoenix, AZ, USA) and Alexeter Technologies (Wheeling, IL, USA) are active in this area. A five-channel commercial colorimetric biosensor on the principle of lateral flow assay is depicted as Figure 1. There also exists optical flow through instruments for the detection of biological warfare agents. Devices Raptor and Biohawk (Research International, Monroe, WA, USA) make automatic fluorometric assay based on the recognition capability of cyanine labelled antibodies. These commercial biosensors are suitable for the assay of staphylococcal enterotoxin B, ricin, botulinum toxin, *Francisella tularensis*, *Escherichia coli* O157:H7, *Yersinia pestis* and *Bacillus anthracis* [39,40,41,42,43,44,45]. Up to four biological agents by Raptor, and up to eight one by Biohawk, can be contemporary analyzed. Biosensor 2200R (MSA, Pittsburgh, PA, USA) is another promising device suitable for the detection of a wide group of biological warfare agents. In its principle, it performs an immunoassay based on magnetic nanoparticles that capture analyte from a sample. Interaction with the fluorescence labelled antibodies is the second step. *Bacillus anthracis*, ricin, botulinum toxin, *Yersinia pestis*, *Francisella tularensis*, *Vibrio cholerae*, staphylococcal enterotoxin B and West Nile virus can be analyzed by the device.

The current research on optical biosensors have two major lines that have impact on their applicability:

- Firstly, new materials for immobilization of biorecognition part of biosensors, unique nanoparticles, improved biorecognition parts of biosensors and optically active materials such as quantum dots make the colorimetric biosensors more competitive [46,47,48,49].
- Secondly, new techniques making optical assays more friendly for practical use have appeared. The colorimetric test based on digital cameras is an example of such techniques [50,51,52,53,54,55,56].

Recently, promising optical biosensors were proposed as a tool for the determination of biological warfare agents using advanced nanotechnologies. The development of methods based on new materials can be seen in the work by Rong et al., who manufactured manganese-doped carbon dots with ethylene diamine and ethylenediamine tetraacetic acid with bound Eu^III+^ [57]. The modified carbon nanoparticles interacted with 2,6-dipicolonic acid, which is a biomarker of *Bacillus anthracis* spores, and the presence of 2,6-dipicolonic acid caused change of fluorescence from intense blue to bright red. The assay exerted linearity from 0.1 to 750 nmol/L and limit of detection was 0.1 nmol/L. The fact that the fluorescence appeared quite immediately after sample application (within 1 min) is another advantage. Photonic crystals, i.e. crystal affecting photon motion, are another nanostructure bringing high application potential into biosensors construction. Zhang et al. prepared photonic crystal with total internal reflection with single stranded DNA captured through biotin-streptavidin interactions and used it for the detection of DNA from *Bacillus anthracis* [58]. The interaction of DNA from a sample with the immobilized single stranded DNA caused resonant wavelength shift. The limit of detection for *Bacillus anthracis* DNA was equal to 0.1 nmol/L. The authors did not provide specification of time per one assay, but considering the samples manipulation and tempering steps, the assay should be finished within 1 h.

The biosensors can be based on long-period fiber gratings covered with a nanostructured film or membrane. Such an approach was made in the work by Cooper et al. for the detection of *Francisella tularensis* [59]. They prepared an optical interferometic sensor with immobilized probe for *Francisella tularensis* subspecies *tularensis* and subspecies *holarctica*. The method exerted a good limit of detection ranging in nanograms of DNA. Sample processing (boiling) lasted 5 min, interaction with sensor another 5 min, and considering other steps (rinsing, spectrum recording)—the assay should be finished within 20 min. The interferometry technique was used also by Mechaly et al. who prepared a biolayer consisting of biotynylated antibodies linked in the presence of analyte with another, phosphatase labeled, antibodies causing deposition of non-soluble crystals causing wavelength interference [60]. The assay was based on Bio-Layer Interferometry based on fiber optic biosensors and standard 96-well microplates, and it was suitable for the determination of *Francisella tularensis* and ricin with a limit of detection for 10^4^ CFU/mL for *Francisella tularensis* and 10 pg/mL for ricin. The authors claimed they finished the assay within 17 min.

Advanced optical methods can serve as a platform for a biosensor construction. An optical microchip with integrated high-precision Bragg gratings is an emerging platform suitable to be modified with antibodies and can serve as a biosensor. This concept was chosen Bhatta for the assay of *Bacillus anthracis*, *Francisella tularensis*, Vaccinia virus and ricin toxin [61]. Surface plasmon resonance is another optical platform providing improved analytical parameters comparing to standard spectral methods. The possibility to perform the assay as label free is the main advantage. On the other hand, the relatively high price of the devices and cost per assay is a disadvantage. The concept of biological warfare agents assay by surface plasmon resonance biosensor can be learned from the work of Leveque et al., who prepared a biosensor for botulinum toxins A and E [62]. The biosensor used the fact that botulinum toxin is an enzyme, and it did not measure botulinum toxin directly, but instead it contained the immobilized antibody against SNAP25 and measured this peptide, which was cleaved by botulinum toxin enzymatic activity. The concept of SNAP25 detection as an enzymatic product of botulinum toxin was also selected for biosensor construction in an experiment by Shi et al. [63]. They prepared a modified fluorogenic SNAP25 linked to graphene oxide and fluorescence resonance energy transfer was measured in the presence of botulinum toxin. The assay provided an excellent limit of detection at 1 fg/mL and quite a long linear range from 1 fg/mL to 1 pg/mL for light chain of botulinum toxin A. The authors claim specificity for botulinum toxin A light chain because of its specificity to the SNAP25-graphene oxide conjugate. The enzymatic activity of botulinum toxin and determining its presence this way can be made by fluorimetry with digital recording of image. This concept is older and it most likely more ready for practical adaptation. It was for instance described in the work by Balsam et al. for botulinum toxin A and SNAP25 containing fluorogenic peptide in a homogenous phase with the sample [64]. Final fluorescence was recorded by a CCD camera. Limit of detection 1.25 nmol/L was achieved. The assay was constructed to cover up to 16 samples in one moment. 

Optical biosensors typically exert good reproducibility, and visual control of the reaction is also typical for many types of assay. On the other hand, there are also common drawbacks when optical biosensors constructed. The standard optical biosensors can be sensitive to light as the color reagents can degrade. The issue of degradation is of course not relevant for non-linear type of optics, which is more robust in this sense, but it is also significantly more expensive. Even though prices of non-linear devices, such as apparatuses for surface plasmon resonance, have dropped their price in the last years, the total costs still reduce their ability to compete with the other methods. including very cheap but quite reliable colorimetric methods—including colorimetric biosensors. Survey of optical biosensors is given in Table 2.

## 5. Electrochemical Biosensors for Biological Warfare Agents Assay

Electrochemistry is another well-known platform suitable for the construction of biosensors. When compared to the optical methods, there is no single decision of which platform is better, and whether optical or electrochemical biosensors should be preferred. While electrochemical biosensors can be sensitive to the interference of redox active compounds such as metal ions or antioxidants, optical biosensors can exert limitation when colored samples are processed. Moreover, some physical and chemical conditions have impact on extinction coefficients of chromogenic reagents. Costs per assay should also be considered. Many electrochemical methods are in this regard dependent on the prices of noble metals and the increase of metal price on global market, which causes augmentation of cost per assay for voltammetric and other noble metal needing methods. The issue of general types of electrochemical biosensors were reviewed elsewhere [60,65,66,67,68,69,70]. The electrochemical biosensors can be based on the principle of antibodies interaction, recognizing of DNA sequence, recognizing metabolic or other activity of the whole cells or use of specific enzymes [71]. Overview of electrochemical biosensors can be learned from Table 3.

**Figure 2:**
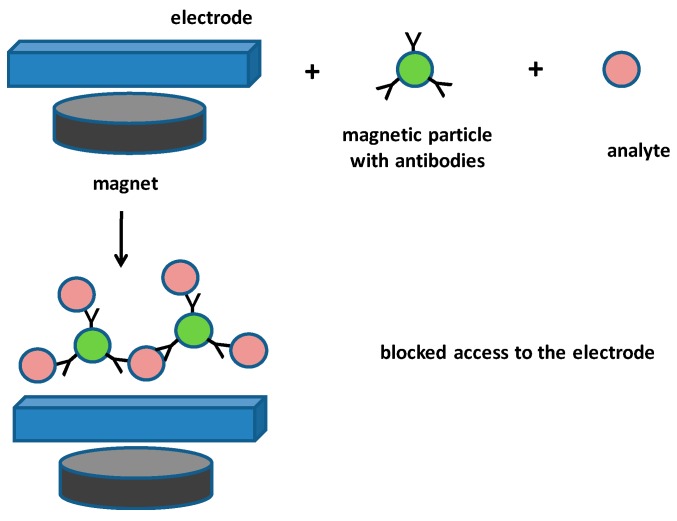
Simplified principle of magnetic particles blocking access to the electrode surface. It is a type of assay as described in the cited work [76].

Voltammetric and potentiometric biosensors have become quite advanced, exerting good analytical parameters such as sensitivity and low limits of detection, as well as having low costs for both the measuring instruments and the disposable material, such as electrodes. The potentiometric biosensors can be constructed on a semiconductor platform as presented below, which makes them quite accessible due to a recent decrease of prices in the manufacturing semiconductors components manufacturing. Regarding the voltammetric, screen printed electrodes can be represented at such low cost, but also as a highly effective platform [49,72,73,74,75]. An example of a screen-printed electrode is shown in Figure 2. The concept of a biosensor based on screen-printed electrodes was for example chosen in the work by Settering and Alocilja [76], who used a screen-printed carbon electrode sensor in combination with magnetic polyaniline nanoparticles modified with an antibody, which catches the analyte, and was then drawn by an external magnet to an electrode surface. Cyclic voltammetry was used to record signals. Voltammograms were differing for nanoparticles with captured analyte and free nanoparticles, because the particles containing the analyte significantly blocked the access to the electrodes and had in this way an impact on the voltammograms. The assay was suitable for *Bacillus cereus* (a surrogate for *Bacillus anthracis*) and for *Escherichia coli* O157:H7. A limit of detection 40 CFU/mL for *Bacillus cereus* and 6 CFU/mL for *Escherichia coli* O157:H7 was reached. The assay was finished within 1 h and the authors claim long term stability one year for the stored biosensors. A simplified principle of magnetic particles in combination with the blocking of access to the electrode surface is depicted as Figure 2.

**Figure 3:**
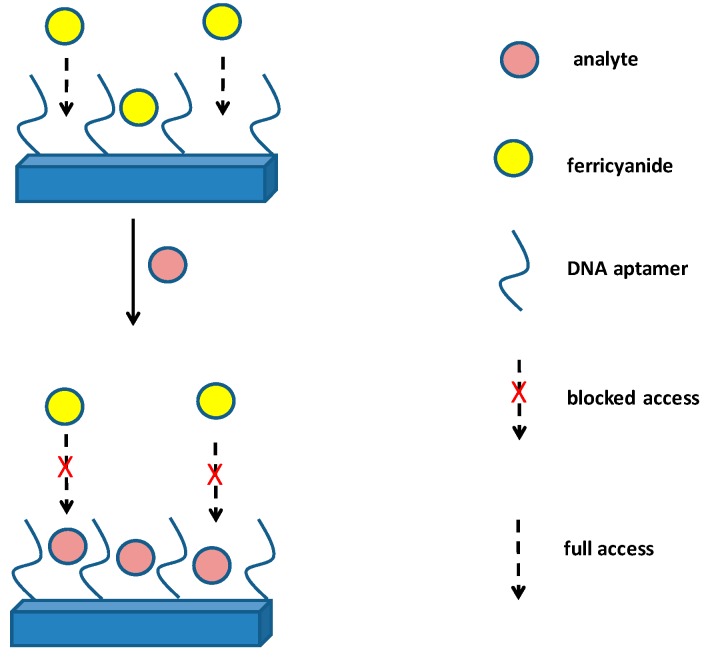
Principle of impedimetric sensor with immobilized ssDNA working on principle when a redox active compound, such as ferricyanide (3-), has limited access to electrode surface. This principle was for instance introduced in the work by Mazzaracchio et al. [77].

An impedimetric biosensor based on gold screen printed electrodes was introduced in the work by Mazzaracchio et al. [77]. The authors used it for the detection of *Bacillus anthracis* simulant *Bacillus cereus*, and the detection was possible due to DNA aptamer recognition potency. The principle of this assay, which is based on blocking ferricyanide access to the electrode surface, is depicted in Figure 3. The assay needed an incubation time of 3 h and it exerted linearity from 10^4^ to 10^6^ CFU/mL and the achieved limit of detection was equal to 3 × 10^3^ CFU/mL. Another DNA recognizing biosensor was developed by Raveendran et al. for the determination of *Bacillus anthracis* [78]. The biosensor contained ssDNA immobilized on modified gold screen printed electrodes. Cyclic voltammetry in the presence of ferricyanide (3-) was measured in the range −0.5–0.7 V and the volammograms differed when DNA fragments from Bacillus anthracis were captured on the electrode surface by interactions with the ssDNA and blocked access of ferricyanide (3-) to the electrode. The system allowed for the detection of 10 pmol/L of DNA from *Bacillus anthracis*. Stability of the biosensor for three months was reported by the authors. The authors did not write time per one assay cycle, but it can be inferred from the longest step of the assay—hybridization—which lasted 1 h. 

An electrochemical biosensor was for instance also developed by Ziolkowski et al. for the detection of *Bacillus anthracis* by recognizing the pagA gene [79]. The biosensor contained a folded DNA molecular beacon probe linked to gold electrodes. In the presence of *Bacillus anthracis,* respective of its pagA gene, the probe became unfolded and electrochemical properties of the modified electrodes are recorded. Limit of detection for the biosensor was 5.7 nmol/L and it exerted a linear range of 22.9–86.0 nmol/L for a 5 min lasting assay. Platforms based on semiconductors are another way to construct a biosensor with applicability to various analytes [80,81,82,83,84,85]. They are also suitable for biological warfare agents, which can be seen from following examples. Choi et al. manufactured a light addressable potentiometric sensor with immobilized antibodies against botulinum toxin, and interaction of the biosensor with botulinum toxin was followed by the application of urease labelled antibodies [86]. This immunoassay based on a light-addressable potentiometric biosensor had a limit of detection of 10 ng/mL. The authors also compared the light-addressable potentiometric sensor platform with surface plasmon resonance and claimed that the light addressable potentiometric sensor assay exerted a better limit of detection than label-free real time assay by a surface plasmon resonance biosensor. The better sensitivity makes the potentiometric biosensor competitive to the standard immunoassay of botulinum toxin.

Magnetic beads and an electrochemically active label on an antibody were chosen as a platform in the work by Cunningham et al. [87]. The researchers prepared magnetic beads covered with antibodies against ricin and another anti-ricin antibodies modified with silver nanoparticles. In the presence of ricin, a complex was formed, containing the both magnetic beads and silver nanoparticles. The complexes were magnetically separated and silver nanoparticles were electrochemically measured. The assay exerted limit of detection for ricin 34 pmol/L within an assay time of 9.5 min. 

## 6. Piezoelectric Biosensors for Biological Warfare Agents Assay

**Figure 4:**
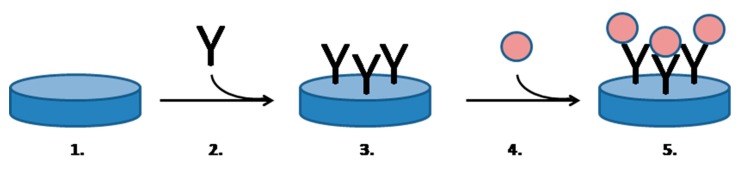
An idealized principle of piezoelectric function. Meaning of numbers: 1. Pure QCM sensor with high frequency of oscillations; 2. Immobilization of an antibody; 3. Finished biosensor prepared for an assay; 4. Application of a sample containing analyte (a biological warfare agent); 5. Analyte is caught on the biosensor surface and frequency of oscillation is decreased.

Piezoelectric biosensors are other platforms suitable for biological warfare agents’ assay. Compared to most of the other types of biosensors; piezoelectric biosensors are generally suitable for a label free assay and it records direct affinity interaction with surface, rather than chemical or physical properties of the medium, such as the other types of biosensors. Common reviews surveying principles and general applications of the piezoelectric biosensors are given in references [19,20,88,89,90,91,92,93]. An idealized principle of a piezoelectric biosensor containing an antibody and directly reacting with analyte is depicted in Figure 4. The major advantage of the piezoelectric biosensors is based on their ability to directly record affinity interactions. Therefore, they can work as label-free devices and directly monitor affinity interactions between hence antibodies or antigens, can be immobilized and can also interact with their counterpart. There is also a possibility to make molecularly imprinted polymers on their surface [94]. Though there are not available analytical devices containing molecularly imprinted polymer as a sensitive part for the determination of biological warfare agents, the technique is advantageous due to simple mass production, which uses simple organic materials.

Piezoelectric biosensors were proposed as a simple tool for biological warfare agents’ detection in several studies. In the work by Poitras and Tufenkji, *Escherichia coli* O157:H7 was detected by a QCM biosensor containing purified polyclonal antibodies onto crystal surface [95]. The authors reported the ability to detect 3 × 10^5^ cells/mL and the biosensor was able to quantify the presence of *Escherichia coli* O157:H7 up to a concentration of 10^9^ cells/mL in a real time assay. In another work, Hao et al. used QCM modified with polyclonal antibody for the detection of *Bacillus anthracis* spores [96]. This biosensor provided a limit of detection of 10^3^ CFU/mL. The assay can be performed in a flow through array and real time results can be achieved. On the other hand, the better and aforementioned limit of detection is achieved when the biosensor is treated by drying. In this case, the assay is finished within 30 min. 

The piezoelectric biosensors can also be used as a diagnostic tool, providing information about disease development, and can help to reveal the misuse of a biological warfare agent when victims occur. Piezoelectric biosensors containing immobilized antigen from *Francisella tularensis* and exerting sensitivity to antibodies were proposed as a tool of fast diagnosis [97,98,99]. In this case, an antibody specific to *Francisella tularensis* was the analyte and it was determined by the biosensor. Overview of piezoelectric biosensors can be seen in Table 3.

## 7. Conclusions

Biosensors for the detection and quantification of biological warfare agents represent a group of analytical tools that have tactical relevance when countermeasures are planned and practical importance when considered their price, simplicity and portability. Though only a small number of biosensors became commercialized, the practical experience from the commercialized one, and the fact that these tools serve as equipment for search teams in the military and other organization making countermeasures against biological warfare agents, are proof of their potential. The recent research on the issue further improved the applicability and practical relevance. It is not expected that the biosensors become a direct competitor to standard methods such as PCR, chromatography or mass spectrometry, but they are the first tool of choice for field research and a reserve for laboratories verifying and supporting the other, more exact, but also more expensive and elaborative, standard methods. The recent progress in biosensors construction makes them more promising for the future. 

The next development of biosensors will be conditioned by availability of new specific materials and their price. The biosensor for biological warfare agents will be depending on the process as well. Quantum dots [46,100,101], magnetic micro and nanoparticles [102,103], screen printed electrodes and other 3D printed materials [75,104,105], electrochemically and fluorimetrically active nanoparticles [106,107,108,109,110,111,112] and advanced sensing materials, such as aptamers [113,114,115,116], can be highlighted as promising material platforms in a brief summarization, considering previous reviewing.

## Figures and Tables

**Table 1:** Survey of important biological warfare agents.

Category According CDC	Biological Warfare Agent	Type of the Agent	Caused Disease
A	*Bacillus anthracis*	Bacterium	Anthrax
	*Francisella tularensis*	Bacterium	Tularemia
	*Clostridium botulinum* including its toxins	Bacterium producing Botulinum toxin	Poisoning by toxin
	*Variola major*	Virus	Smallpox
	Marburg	Virus	Marburg hemorrhagic fever
	Lassa	Virus	Lassa hemorrhagic fever
	Machupo	Virus	Bolivian hemorrhagic fever
B	*Burkholderia mallei*	Bacterium	Glanders
	*Burkholderia pseudomallei*	Bacterium	Melioidosis
	*Brucella melitensis*	Bacterium	Brucellosis
	*Chlamydia psittaci*	Bacterium	Chlamydiosis
	*Escherichia coli* O157:H7 including its shiga toxins	Bacterium	Foodborne illness, poisoning by shiga toxin
	*Rickettsia prowazekii*	Bacterium	Typhus
	*Vibrio cholerae*	Bacterium	Cholera
	*Staphylococcus aureus* including its toxins	Bacterium producing a group of staphylococcal enterotoxins	Staphylococcal infections, Poisoning by staphylococcal enterotoxins
	Ricin	Toxin from a plant *Ricinus communis*	Poisoning by ricin

**Table 2:** Optical biosensors and bioassays for biological warfare agents assay.

Analyte	Principle	Specific Material in Biosensor	Limit of Detection	Other Specifications	Reference
2,6-dipicolonic acid—a marker of *Bacillus anthracis*	The modified dots interacted with 2,6-dipicolonic acid; it resulted in change of fluorescence color	Manganese-doped carbon dots with ethylene diamine and ethylenediamine tetraacetic acid with bound Eu^III^	0.1 nmol/L	Results within 1 min	[57]
DNA from *Bacillus anthracis*	Photonic sensor immobilized single stranded DNA; interaction with DNA from sample causes resonant wavelength shift	Photonic crystal sensor with total-internal-reflection modified with DNA	0.1 nmol/L	Results within 1 h	[58]
DNA from *Francisella tularensis*	Optical inteferometry using DNA probes	Long-period fiber gratings	1 ng	Results within 20 min	[59]
*Francisella tularensis* and ricin	Optical inteferometry using immobilized antibodies and antibodies labeled with alkaline phosphatase—the enzyme finally caused a deposition of insoluble crystals, which was measured by the interferometry	Bio-layer interferometry based on fiber optic biosensors and standard 96-well microplates	10^4^ CFU/mL for *Francisella tularensis* and 10 pg/mL for ricin	Results within 17 min	[60]
Botulinum toxin A	Botulium toxin converting fluorogenic peptide containing SNAP25 precursor located on graphene oxide, fluorescence resonance energy transfer is measured	Graphene oxide modified with a peptide	1 fg/mL	Selective for light chain of Botulinum toxin A	[63]
Botulinum toxin A	Botulium toxin convert fluorogenic peptide containing SNAP25 precursor, fluorescence is measured by CCD photodetector	Fluorogenic peptide	1.25 nmol/L	Assay of 16 samples contemporary	[64]

**Table 3:** Electrochemical and piezoelectric biosensors and bioassays for biological warfare agents’ assay.

Analyte	Type of Biosensor	Principle	Specific Material in Biosensor	Limit of Detection	Other Specifications	Reference
*Bacillus cereus* and *Escherichia coli* O157:H7	Voltammetric	Cyclic voltammetry on screen printed electrodes; analyte was captured and magnetically separated by magnetic nanoparticles; voltammograms were differing due to the interaction.	Polyaniline/magnetic immunoparticles	40 CFU/mL for *Bacillus cereus* and 6 CFU/mL for *Escherichia coli* O157:H7	Results within 1 h, stability of storage biosensors for at least 1 year	[76]
*Bacillus anthracis*	Voltammetric	Electrode contains DNA molecular probe for Bacillus anthracis; in its presence, electrochemical properties of the electrode changed.	Gold electrode modified with genetic probe	5.7 nmol/L	5 min lasting assay	[79]
*Bacillus cereus*	Impedimetric	DNA aptasensor interacts with specific sequences in *Bacillus cereus*, impedimetry on screen printed gold electrodes is measured	Screen printed gold electrodes with DNA aptamer	3 × 10^3^ CFU/mL	Incubation time 3 h is necessary for the assay	[77]
*Bacillus anthracis*	Voltammetric	Electrodes contained immobilized ssDNA, it interacted with DNA fragments from *Bacillus anthracis*, cyclic voltammetry was performed and voltamograms were differing due to the interactions	Gold screen printed electrode modified with DNA	10 pmol/L	The longest step of the assay (hybridization) lasted 1 h; reported long term stability of the biosensor for 3 months	[78]
Botulinum toxin	Potentiometric	Potentiometric electrode covered with antibodies, the assay was a sandwich format principle based on secondary urease labelled antibodies	Light addressable potentiometric sensor	10 ng/mL	The light addressable potentiometric sensor assay exerted better limit of detection than label-free real time assay by a surface plasmon resonance biosensor	[86]
Ricin	Voltammetric	Antibody modified magnetic beads and silver nanoparticles also covered with an antibodies formed complex with ricin; the complex was magnetically separated due to the magnetic nanoparticles and it was also electrochemically active due to the silver nanoparticles.	Magnetic beads covered with antibody, silver nanoparticles with antibody	34 pmol/L	9.5 min lasting assay	[87]
*Escherichia coli* O157:H7	Piezoelectric	Piezoelectric biosensor with antibodies against *Escherichia coli*, affinity interaction is measured piezoelectrically.	QCM	3 × 10^5^ cells/mL	Real time assay	[95]
*Bacillus anthracis*	Piezoelectric	Piezoelectric biosensor with polyclonal antibodies against *Bacillus anthracis*, affinity interaction is measured piezoelectrically.	QCM	10^3^ CFU/mL	30 min lasting assay	[96]
